# A Comprehensive Evaluation of the Genetic Relatedness of *Listeria monocytogenes* Serotype 4b Variant Strains

**DOI:** 10.3389/fpubh.2017.00241

**Published:** 2017-09-13

**Authors:** Laurel S. Burall, Christopher J. Grim, Mark K. Mammel, Atin R. Datta

**Affiliations:** ^1^Center for Food Safety and Applied Nutrition, U.S. Food and Drug Administration, Laurel, MD, United States

**Keywords:** *Listeria monocytogenes*, serotype 4b variant strains, WGS comparisons, multi-virulence-locus sequence typing, WGS SNP analysis

## Abstract

Recently, we have identified a link between four listeriosis incidents/outbreaks to a variant of *Listeria monocytogenes* (Lm) serotype 4b strains, 4bV. Although 4bV strains have been reported from clinical specimens as well as from foods, listeriosis outbreaks occurring in 2014–2016 were the first reported outbreaks involving 4bV in the USA. Since traditional typing methods do not detect members of this group, we undertook a systematic and retrospective analysis of all Lm in the NCBI WGS Sequence Read Archive database to investigate the burden of 4bV strains among all listeriosis cases. This analysis identified the presence of isolates causing sporadic cases as well as those associated with the aforementioned outbreaks, as determined by WGS and traditional epidemiology. In total, approximately 350 Lm 4bV strains were identified from multiple parts of the USA as well as from Australia and Chile, dating back to 2001. The genomic relatedness of these strains was compared using the CFSAN SNP Pipeline and multi-virulence-locus sequence typing (MVLST). Using the CFSAN Pipeline tool, the 4bV strains were found to group into seven clusters that were separate from 4b strains. All seven clades appeared to contain isolates from both clinical and non-clinical sources. Conversely, the MVLST analysis revealed that practically all of the strains belonged to a single clade, suggesting that 4bV strains from disparate geographic regions and sources are under varied selective pressure, restricting diversity across these six virulence loci while allowing more variability across the genome as a whole. Further evaluation of these 4bV strains identified genes potentially acquired from a lineage II source external to the lmo0733–lmo0739 region, as well as highly conserved SNPs unique to the 4bV strains when compared to those from other lineages. Taken together, these data suggest that 4bV strains have undergone adaptive responses to selective pressures that may enhance survival in the environment while maintaining the pathogenic potential of serotype 4b strains.

## Introduction

*Listeria monocytogenes* (Lm) is a Gram positive, facultative anaerobe that is ubiquitous in nature and responsible for two forms of listeriosis: gastroenteritis and invasive infection. The latter is a comparatively rare foodborne disease with a defined at-risk group including the elderly, pregnant women, and their neonates, as well as immune-compromised individuals, causing about 1,600 cases in the USA each year, leading to about 250 deaths (1). While the case incidence is low, invasive listeriosis has a significant impact on public health due to the high rate of hospitalization (>95%), high mortality rate (15–20% even with treatment), and long-term morbidity due to the nature of the central nervous system syndromes it causes (2). Although there are 13 recognized serotypes for Lm, only four of them are of significant concern with three, 1/2a, 1/2b, and 4b being responsible for >95% of invasive listeriosis cases (2). Historically, the majority of the clinical cases and outbreaks are associated with serotype 4b strains, while 1/2a and 1/2c strains are more typically associated with food and environmental samples (3, 4). These serotypes mostly correlate with distinct lineages (I, II, III, and IV) as described elsewhere (5). Recent reports of several listeriosis cases have highlighted the possible emerging or expanding role of a variant of serotype 4b, detected with a PCR method initially developed by Doumith et al. (6), in recurring outbreaks and sporadic cases (7–9). This variant, termed 4bV or IVb-v1, is serologically identical to 4b strains but contains a 6.3 kb segment of DNA normally restricted to lineage II (1/2a, 3a, 1/2c, and 3c) strains (10, 11). This region contains six genes, lmo0734–lmo0739, that are unique to lineage II strain, although several loci with very limited homology are found in 4b strains for all but lmo0737. Based on motif analyses, these genes encode a transcriptional regulator (lmo0734), a putative ribulose-5-phosphate 3-epimerase (lmo0735), a putative ribose-5-phosphate isomerase (lmo0736), phosphotransferase system IIABC (lmo0738), and a glucosidase (lmo0739). The protein encoded by lmo0737 has no annotated function. Prior work generating a deletion of this region in EGD-e, a laboratory-adapted 1/2a strain, found no corresponding phenotype in the assays tested, including evaluation of ribose metabolism, leaving the role of this region in Lm metabolism still unknown (12).

Evaluation of 4bV strains associated with four recent listeriosis incidents highlighted a high degree of relatedness among these strains (8). Further, epidemiological data identified clinical cases caused by these variant strains dating back to 2003 with a sudden increase in incidence as evidenced by the four listeriosis events: two large outbreaks, a large stone fruit recall linked to two clinical cases and a cluster of cases potentially linked to a cheese product (8, 13–15). These events were all linked or likely linked to foods from a region of California, raising concerns about either endemic contamination or cross-contamination due to some standard agricultural processes or sources (8).

The recent detection of listeriosis cases associated with 4bV strains raises the questions as to what proportion of all reported clinical cases of listeriosis are due to infections with this group of strains, and what factors account for an apparent increased incidence of outbreak-related infections with 4bV strains. As 4bV strains contain the genomic backbone of serotype 4b strains, along with the acquisition of a DNA segment from lineage II strains, which have a bias for food and environmental contamination, the possibility exists that 4bV strains could represent the emergence of a new group with altered virulence and/or environmental adaptation enhancing persistence and/or transfer. From an epidemiological perspective, potential cross-contamination between the firms involved in these recent outbreaks (8, 13–15) suggests that 4bV strains are inherently less susceptible to standard sanitation procedures. Reports of outbreak-related cases of listeriosis among young, healthy individuals and their links to 4bV strains raise the possibility of altered virulence potential of these organisms. These individuals were either too young for pregnancy to be a factor or were male and all were at least 3 years old (14, 15). Given these observations, we interrogated WGS sequences available at NCBI for the presence of additional 4bV strains, to determine the true burden of this emerging variant and to understand the genetic diversity within this group.

## Materials and Methods

### Genome Identification

A list of candidate Sequence Read Archive (SRA) files was pulled from several NCBI BioProjects (*n* = 34) listing Lm as their focus. These SRA files were evaluated using BLAST with query sequences derived from ORF2110, specific to 4b strains, and lmo0737, linked to the 6.3 kb segment, the genomic signatures of 4bV strains (3, 6, 11). SRA files yielding matches for both targets, ORF2110 and lmo0737, were downloaded for further evaluation within this work. Strains identified in prior work were also included in this study (8). The strains used in this study along with the available metadata are presented in Table S1 in Supplementary Material.

### Genomic Comparisons

Sequence Read Archive files were analyzed using the CFSAN SNP Pipeline, and resulting SNP alignment files were used to generate phylogenetic trees utilizing the maximum likelihood method (with bootstrapping, *n* = 1,000) within the MEGA7 software (16, 17). SNP distance matrices were also obtained and used to quantify differences between and within the various subsets. Additional isolates including reference strains, 4b strains from sources that were isolated during the same time frame as 4bV strains or linked to the incidents were added to the dataset.

In addition, the sequences linked to the six virulence genes were extracted from WGS datasets and used in multi-virulence-locus sequence typing (MVLST) analysis (18). For MVLST analysis, sequences for the six genes (*clpP, lisR, prfA, dal, inlB*, and *inlC*) were concatenated, aligned by ClustalW and used to generate phylogenetic trees using the maximum likelihood method (with bootstrapping, *n* = 1,000) in MEGA7 (16, 17).

### SNP Comparisons

The region encoding the acquired 6.3 kb genetic island as well as 500 bp of flanking sequence, on both sides of the island, was identified and extracted from WGS datasets from a collection of strains including serotype 4a, 4b, 4bV, 4c, 4d, 1/2b, 1/2a, 1/2c, 3a, and 3c, and these sequences were aligned using MEGA7 (16). Non-conserved bases were identified and compared across the strains, and the percent occurrence of each SNP within each serogroup was calculated to determine the prevalent SNP. These data were compared to assess whether individual bases in the region of 4bV strains matched lineage I or lineage II strains or if they represented an SNP unique to 4bV strains.

### Genome-Wide Comparisons

Five strains, one for each of the three largest 4bV clades, one 4b isolate from the caramel apple outbreak (14) (FDA00008715/SRR1763833), and a 1/2a isolate unlinked to this study (LS884), were assembled using CLC Genome Workbench 7 (Qiagen), annotated in RAST, and compared in the SEED Viewer comparative genomics tool, using LS884 as the reference genome (19). The strains selected to represent the three 4bV groups were PNUSAL000137 (SRR974868) for Clade 1, PNUSAL000097 (SRR972399) for Clade 2, and PNUSAL000136 (SRR974867) for Clade 5. BLAST percent identities were compared to identify genes present in the 4bV isolates that were divergent or missing from the 4b isolate and present in the 1/2a isolate. A BLAST-based comparison of the 4bV strains to the apple 4b isolate as the reference strain was also performed to verify the absence of an ortholog in the 4b strain. We eliminated loci that had a difference of less than 10% identity for the various comparisons, greater homology to the 4b reference strain, and those loci with >90% identity in all the genomic comparisons.

## Results

### Identification and Characteristics of 4bV Strains

A total of 6,830 Lm SRA files were identified for BLAST evaluation. Of these, 400 were positive for ORF2110 and lmo0737, a rate of about 5%, which is higher than previously observed (11). Several of these were duplicate SRA entries for the same strain (duplicate NCBI Biosamples). In addition, BLAST comparisons of the lmo0737 and ORF2110 genes against the assembled genome identified several false positives (~12% of initial candidates). These false positives were likely due to the low level of sequence homology required for the initial candidate identification and were not included in the final dataset, with one exception. Strain PNUSAL001558 (SRR2134936) was positive for ORF2110 and lmo0737 but was negative for a second 4b target, ORF2819 (6). It was also negative for a second 1/2c target, lmo1118; therefore, this strain could not be serotyped using the multiplex PCR method (6). Due to its novelty and indeterminate serotype, the isolate was retained in the dataset.

Additional whole-genome sequences from the prior analyses of four 4bV-linked incidents (4, 8) as well as sequences from strains recently received in our laboratory were also included in this study. In total, we identified 387 strains to compare, comprised 20 4b and 367 4bV strains, the latter being confirmed as 4bV by *in silico* or PCR analysis. As 29 of the stone fruit isolates were derived from our laboratory during enumeration studies of the stone fruits (20), we excluded six of these strains from the analysis to reduce overrepresentation of these isolates, although they are noted in Table S1 in Supplementary Material. The excluded strains were chosen at random from the different lots of product tested while ensuring that all lots were still represented within the dataset, and prior analysis has shown these isolates to be highly related (9). It should be noted that other strains in the dataset are also likely to be over-represented as they are associated with investigations, leading to biased isolate collection. However, given these strains can represent diverse environmental sources in a production facility or lacked product lot information, we did not exclude any strains from other sources to ensure completeness. This resulted in a final WGS dataset of 361 4bV isolates, including 4 strains from Australia, 1 from Chile, and 350 from the USA with strains from 16 different states, although some of these may be linked to food from other states due to transport of food products (Table S1 in Supplementary Material). 4bV strains can be found in this collection as far back as 2001, although the majority (*n* = 286) are from after 2013, the start of GenomeTrakr (21).

Examination of the sources of the 4bV strains showed that the vast majority (*n* = 212) have clinical designations, although 34 of these are simply noted as “clinical/host-associated” which is not a clear attribution. In addition, there is a bias toward older patients with 76% of them over the age of 49 (62% over 60). Conversely, there are only 12 patients under the age of 20, with three clearly falling outside of the neonates group. Evaluation of the outbreak reports also indicated another patient in the range of 0–4 years was 3 years old (15). This leaves 12% of the cases as adults between the ages of 20–49, which is the age bracket that includes most pregnant women although not all of these patients were pregnant. This shows a strong bias toward infections affecting the elderly, consistent with recent trends in listeriosis involving all other serotypes (22–24).

We found 67 4bV isolates that were collected from environmental samples, mostly from production facilities, although two were from soil samples. The remaining samples were from food products including atypical foods (apples, lettuce, collard greens, curly parsley, nectarines, peaches, spinach, and walnuts) and the more typical foods (seafood, sprouts, deli meat, and cheese). The foods considered “typical” are ones that have been historically associated with listeriosis, while atypical foods are ones only recently associated with listeriosis (22). The higher incidence of 4bV strains in apples and stone fruits is at least partially due to higher sampling of the implicated products during the incidents.

### SNP Distance Analysis

The CFSAN SNP Pipeline generated an alignment file that identified a total of 36,772 variable nucleotides and SNP distance matrix that were used to establish the level of relatedness (Data Sheet S1 and Table S2 in Supplementary Material) among the strains used in this study. Overall, eight clades, one clade comprised the 4b isolates and seven comprised the 4bV isolates, were identified. For the 4b clade, a total of 20 4b isolates, 19 from the apple outbreak and 1 isolate from the Jalisco cheese outbreak in 1985 (25), were included in this portion of the study. These isolates clustered within 200 SNPs of each other, matching prior results (Table S2 in Supplementary Material, green box) (9). In addition, they were divergent from all of the 4bV strains, with SNP distances greater than 8,800. In fact, each of the identified clades is separated from the others by at least 8,000 SNPs. Four of the 4bV clades (Clades 3, 4, 6, and 7) were comprised very few isolates (*n* = 2–4), suggesting these groups may represent rare isolates. This may be due to sampling bias or because these strains are less common. Clades 3 and 6 (Table S1 in Supplementary Material) consist of only two strains each and while another two clades (Clades 4 and 7) have three strains each, although Clade 7 has a fourth isolate that diverges from this clade by about 1,400 SNPs, suggesting a shared common ancestor. Clade 4 has been previously identified, encompassing three of the Australia 4bV strains (3, 10). Clade 6 may represent linked samples but the metadata are too limited to determine this (SNP distance = 17). The other two, Clades 3 and 7, represent strains that are clearly from unrelated sources but are too divergent to support their designation as a clonal group. These clades, Clades 3, 4, 6, and 7, are not further considered in this work given the small sample set for each group, although it should be noted they provide a measure of the diversity of 4bV lineages. It is possible that each of these clades represent independent gene transfer events for the 6.3 kb DNA segment, although we cannot rule out the possibility of some having diverged from a single common, although distant, ancestor.

The remaining three clades (Clades 1, 2, and 5) are all larger, with two of them, Clades 1 and 2, representing the dominant 4bV clades observed in this study. Some of the overrepresentation is likely due to repeated isolation of certain strains during focused foodborne outbreak incident investigations. Clade 1, previously identified by Burall et al. (8), is composed of isolates separated from each other by fewer than 200 SNPs and is comprised 163 isolates (Table S2 in Supplementary Material, violet box). Clade 1 is split into four subclades (1a–1d) along with outliers, simply denoted as Clade 1 (Table S1 in Supplementary Material, Figure 1). These subclades roughly correspond with four incidents previously described (8, 9). However, these groups are all very tightly related to each other when compared to the diversity seen in Clade 2.

**Figure 1:**
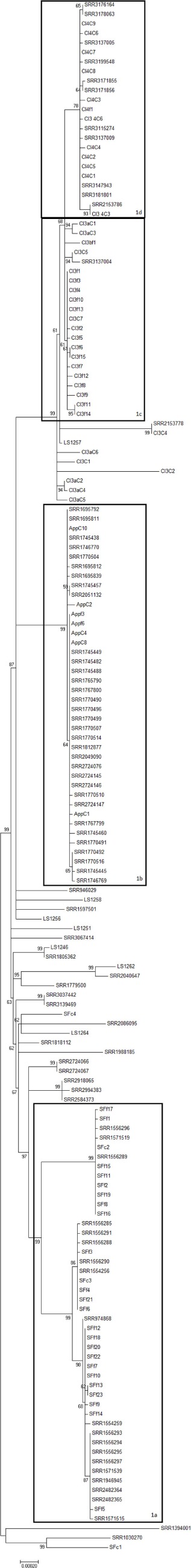
A maximum likelihood tree of members of 4bV Clade 1 derived from SNP alignment file generated using the CFSAN SNP Pipeline with bootstrapping (*n* = 1,000). Strains linked to specific outbreaks are renamed as noted in Table S1 in Supplementary Material for ease of reference. Subclades within Clade 1 are indicated. The scale bar indicates distance as assessed by the Tamura–Nei method.

**Figure 2:**
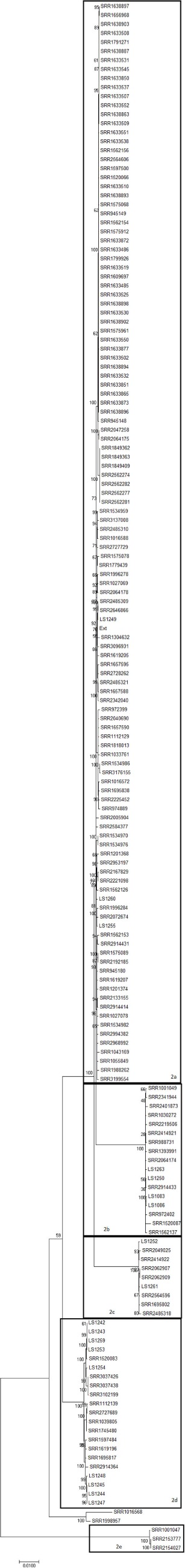
A maximum likelihood tree of members of 4bV Clade 2 derived from SNP alignment file generated using the CFSAN SNP Pipeline with bootstrapping (*n* = 1,000). Strains linked to specific outbreaks are renamed as noted in Table S1 in Supplementary Material for ease of reference. Subclades within Clade 2 are indicated. The scale bar indicates distance as assessed by the Tamura–Nei method.

Clade 2, which contains 172 strains, is comprised five subclades (2a–2e) (Figure 2; Table S1 in Supplementary Material). The subclades in Clade 2 differ from each other by as much as ~4,500 SNPs but show a continuum of SNP distances with no clear demarcation of separate ancestry. Further, SNP differences of over 9,000 were observed between Clade 2 and non-Clade 2 strains (Table S2 in Supplementary Material, orange box). In addition, these isolates form a clear cluster on the complete 4bV phylogenetic tree (Figure S1 in Supplementary Material). Closer evaluation of this clade supports the idea that it likely arose from a single common ancestor but has undergone greater observed diversification than Clade 1 (Figures 1 and 2).

**Figure 3:**
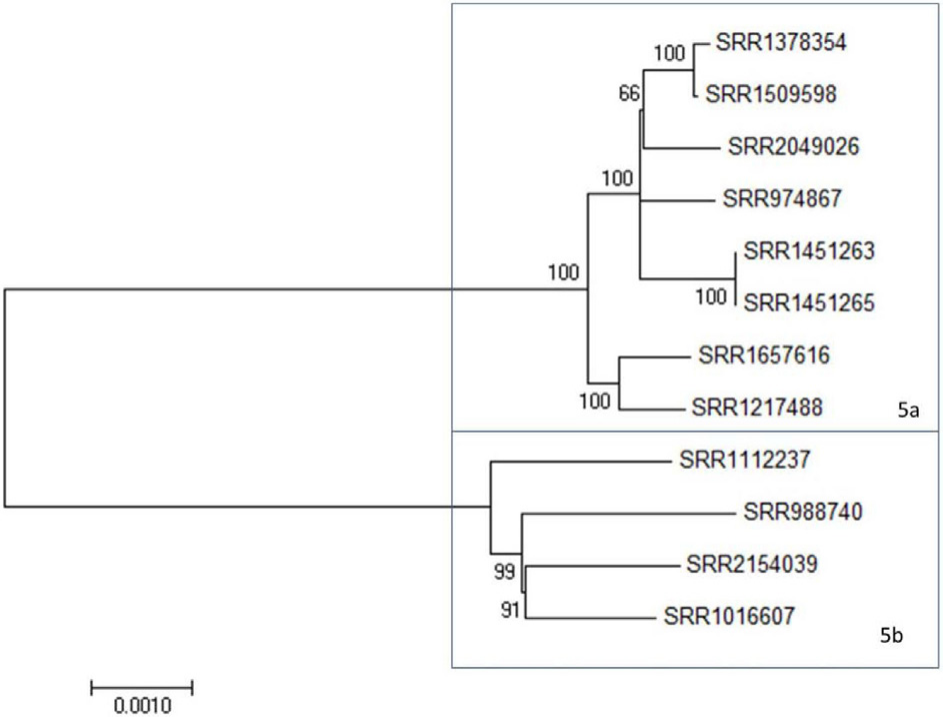
A maximum likelihood tree of members of 4bV Clade 5 derived from SNP alignment file generated using the CFSAN SNP Pipeline with bootstrapping (*n* = 1,000). Subclades within Clade 5 are indicated. The scale bar indicates distance as assessed by the Tamura–Nei method.

In addition to the two large clades, there is a clade of 12 strains, Clade 5, that shows an intermediate level of diversity, compared to the Clades 1 and 2 (Figure 3; Figure S1 in Supplementary Material). Clade 5 is comprised two subclades at <600 SNP differences (Table S2 in Supplementary Material, blue box). All three of these clades include clinical isolates and, with the exception of Clade 2, show strong relatedness when assessed by SNP distances. Clade 2, however, appears to have been derived from a common ancestor but has undergone further divergence than that observed in the other clades to date. Maximum likelihood trees derived from the above data support these observations (Figure S1 in Supplementary Material; Figures 1–3). These three clades provide an opportunity to evaluate 4bV strains from different genomic backgrounds to evaluate potential differences in virulence and adaptation.

### MVLST Comparison

Previously, we reported that strain LS542 (Ext) clustered with strains from Clade 1 by the six gene MVLST approach, although JSpecies tetramer and SNP distance analyses showed clear divergence (8). We examined the strains in this study as well as several reference strains (Table 1), using the MVLST approach, which involved a larger and more diverse set of 4bV strains than the previous study. We found that 4bV strains, which formed seven clusters by the SNP analysis, clustered into one group by the MVLST analysis. Evaluation of these strains, utilizing the six genes MVLST (2,649 bp), revealed a total of 185 SNP locations. The maximum likelihood tree constructed from this alignment shows typical clustering as seen with other MVLST and WGS phylogenetic analyses for non-4bV strains (7, 18, 26). This, however, is not what is observed for the 4bV strains. Instead, certain distinct subclade groups from WGS SNP analysis, specifically Clades 1, 2a, 2b, 2c, and 2e, were collapsed into a single cluster, while WGS SNP Clades 2d, 5, 6, and 7, formed a separate, but more diverse, cluster (Figure S2 in Supplementary Material).

**Table 1:** Reference strains used in the multi-virulence-locus sequence typing analysis (7, 18, 26).

Strain name	Serotype	Clade
FSL C1-387	1/2a	n/a
EGD	1/2a	ECVII
F6854	1/2a	ECIII
FSL J1-101	1/2a	ECIII
L2626	1/2a	ECVII
L2676	1/2a	ECVII
Lm08_5578	1/2a	ECVII
Lm08_5923	1/2a	ECVII
FSL R2-499	1/2a	ECIII
SLCC5850	1/2a	ECVII
FSL J1-194	1/2b	n/a
Finland1988	3a	n/a
SLCC2479	3c	CC9
07PF0776	4b	CC4
Clip80459	4b	CC4
F2365	4b	ECI
H7858	4b	ECII
HPB2262	4b	ECIV
FSL J1-108	4b	ECI
FSL J1-110	4b	ECI
FSL J1-116	4b	ECIV
FSL J1-119	4b	ECI
FSL J1-220	4b	ECIV
J1776	4b	ECII
J1816	4b	ECII
J1817	4b	ECII
J1926	4b	ECII
L312	4b	CC4
LL195	4b	ECI
FSL N3-013	4b	ECIV
Scott A	4b	ECIV
ATCC19117	4d	ECIV
FSL-R2-561	1/2c	
HCC23	4a	
N1011A	1/2b	
SLCC 2376	4c	
SLCC 2540	3b	

We examined the alignment generated by the MVLST analysis (Data Sheet S2 in Supplementary Material) to look at the number of SNP differences between the three largest 4bV clusters and members of epidemic clone (EC) I (27), a previously established clade with strong representation in both of our analytical approaches. We then compared those differences to the average SNP distance seen in the WGS SNP pipeline analysis. We also excluded single incident SNPs from this analysis as 84% (42 of 50) were linked to a single strain (FSL J1-110) and all but one of the remaining to a second strain (SRR1556285). This left 18 SNP locations within the MVLST alignment for ECI, Clade 1, Clade 2, and Clade 5 strains. When ranked by pipeline SNP differences, comparisons of Clades 1, 2, and 5 had a higher average SNP distance between each other than when any of them were compared with ECI strains (Table 2). However, the reverse was seen for the MVLST analysis with the highest diversity observed when comparing 4bV strains to ECI and lesser diversity when compared with each other. Members of subclades 2a, 2b, 2c, and 2e, differed from Clades 1 by 2 MVLST SNPs while the whole-genome SNP differences for the ECI comparisons were higher, despite the WGS differences being roughly equivalent (Table 2). Intriguingly, this loss of apparent diversity is not as dramatic for members of Clades 2d and 5, although these differences still seem small when compared with the whole-genome SNP Pipeline analysis. Analysis for Clade 2 was complicated as one of its subgroups, Clade 2d, differed at several points from the other members of the group, suggesting alternate selective pressures are involved for this subset. Unfortunately, the available metadata for this group is insufficient for identifying possible explanations for this altered diversity. It should be noted, however, that the vast majority of these isolates (10 of the 12) are clinical.

**Table 2:** SNP distances between clades ECI and 4bV Clades 1, 2, and 5 as determined by the CFSAN SNP Pipeline and multi-virulence-locus sequence typing (MVLST).

Compared clades	SNP pipeline range of difference	SNP pipeline average difference	MVLST
ECI vs Clade 1	9,258–9,761	9,660 ± 73	8
ECI vs Clade 2	9,494–10,245	9,482 ± 117	6 (4 for 2 days)
ECI vs Clade 5	8,884–9,174	9,058 ± 56	6
Clade 1 vs Clade 5	10,061–10,629	10,455 ± 98	4
Clade 2 vs Clade 5	9,762–10,604	10,084 ± 105	6
Clade 1 vs Clade 2	9,279–10,157	9,913 ± 122	2 (4 for 2 days)

*The range of WGS SNP distances and average WGS SNP distances with SD were calculated using the SNP difference matrices generated by the pipeline*.

### Evaluation of the 4bV Acquired Region

An alignment of the lmo0733–lmo0739 region was generated in MEGA7 and used to identify SNPs that showed altered representation in the 4bV strains. A total of 269 SNPs were identified; however, those SNPs present in over 80% of the three strain groups, lineage I, lineage II, and 4bV, were removed as showing no bias, leaving 191 SNPs. Ninety-four of these SNPs were within the region absent from lineage I strains, and all but 28 of this subset were present in over 80% of the 4bV and lineage II strains. These 28 SNPs showed conserved substitutions unique to the 4bV strains not present in the lineage II strains.

For the SNPs in the flanking regions shared by all three groups, lineage I, lineage II, and 4bV (*n* = 97), a clear pattern emerged with a transition from a lineage I bias to a lineage II bias and back, as expected by the recombination event (Table S3 in Supplementary Material). Ten SNPs in the flanking region of the 4bV strains showed altered prevalence compared to what was expected based on the bases seen in the typical lineage I and II strains (Table S3 in Supplementary Material). Two of these SNPs showed a reduced frequency of the dominant allele seen in the lineage I and II strains. One had an allele at a higher frequency than observed in the lineage I and lineage II strains. Another six of these 4bV-linked flanking region SNPs showed pronounced reduction (<5%) of the allele observed in the other two groups. The last SNP appeared to be completely random.

Given the presence of SNPs unique to the 4bV group, the protein sequences encoded by the region was evaluated for the presence of amino acid substitutions in the 4bV strains when compared to the lineage II strains. Substitutions were observed for several of the proteins, although most were conservative. Lmo0734 had a shift from 100% threonine to approximately half possessing a serine residue at position 240. Lmo0735 had a near complete (99%) transition from lysine to arginine at position 215. Lmo0737 in 4bV had serine in all but one strain, instead of a proline, at site 237. Lmo0738 had four amino acid substitutions: alanine to valine at amino acid 40, valine to isoleucine at 470, asparagine to serine at 483, and leucine to isoleucine at position 538. Lmo0738 had two amino acid substitutions, glutamate to glycine at amino acid 134 and asparagine to serine at 456.

Most intriguing, however, was the comparison of residues in Lmo0740 among the various strains. Twenty of the 4bV strains had an amino acid composition in this protein observed only in the three lineage III/IV strains, which have a different distribution than the other two lineages (5, 28, 29). This amino acid sequence had several marked differences from that observed in the other 4bV strains, as well as the lineage I and II strains. In total, there were 15 amino acid substitutions in the 227aa Lmo0740 protein (Table 3). Five of these changes resulted in charge changes (positions 21, 23, 25, 42, and 96) while a sixth changed a proline (position 2) to serine. In addition, a change unique to this 4bV subset changes a glycine at amino acid 10 to an aspartate, resulting in another charge change. While it is unclear what effects these changes would have on the final protein, comparison of the two protein version using the NCBI CD search tool finds slightly reduced homology to the CAP binding region with possible changes predicted in putative ligand binding sites, a flexible hinge region, a protein binding region, and a polynucleotide binding region. It is unclear at this point what affect the amino acid changes observed in the other proteins coded for by the genes in this region might have, although the conserved nature of these substitutions across highly divergent clades suggests that they may have a functional role.

**Table 3:** Amino acid substitutions in Lmo0740 between multiple lineages and subsets of *Listeria monocytogenes*.

Position	4bV and lineages I and II	Lineages III and IV	4bV subset
2	Proline	Serine	Serine
10	Glycine	Glycine	Aspartate
21	Tyrosine	Histidine	Histidine
23	Threonine	Lysine	Lysine
25	Lysine	Glutamine	Glutamine
29	Valine (4bV, Lin. I); leucine (Lin. II)	Methionine	Methionine
37	Aspartate	Glutamate	Glutamate
42	Serine	Histidine	Histidine
46	Isoleucine	Valine	Valine
47	Valine	Leucine	Leucine
74	Valine	Isoleucine	Isoleucine
96	Histidine	Serine	Serine
98	Methionine (some Lin. II valine)	Valine	Valine
160	Threonine	Serine	Serine
165	Arginine	Lysine	Lysine

### Genomic Comparisons

To understand what other genomic changes have occurred in 4bV strains, similar to the gain of the 6.3 kb genomic island from lineage II, whole-genome comparative genomic analyses were performed, as described in the Section “Materials and Methods.” This identified 88 genetic loci that comprise the variable genome within this group of strains (Table S4 in Supplementary Material). Among this set were seven genes that encompassed lmo0734 to lmo0739 plus the upstream gene lmo0732, and included lmo0737 which is the only gene without an ortholog in the 4b strain. As these genes were part of or near the site of known recombination they were not further evaluated.

Comparisons of the 4bV strains with the 4b and 1/2a strains identified five loci that were present in all three 4bV strains and the 1/2a reference but absent in the 4b strain. It should be noted, however, that two of the 1/2a genes matched to the same 4bV gene with limited identity to both (57–59 and 68–74%) (Table S4 in Supplementary Material). An additional 39 ORFs present in LS884, the 1/2a strain, lacked an ortholog in the 4b strain, with the determination of presence versus absence defined by a percent identity threshold of 60%. These 39 ORFs were found to have an ortholog in at least 1 of the 4bV representative strains with 8 of these 1/2a loci present in 2 4bV strains (Table S4 in Supplementary Material). However, in seven instances, two or three of the 1/2a genes were found to have homology to a single gene in the 4bV strains. For example, 1/2a genes 121 and 525 (Table S4 in Supplementary Material) were both found to match with high identity (>90%) to the same locus in two of the 4bV strains, suggesting a possible duplication in the 1/2a strain. In the other two instances (1/2a genes 126, 127, and 128 and 1/2a genes 144 and 2768), the homology was found to be higher between one of the 1/2a loci and alternate 4bV strains. For example, 1/2a gene 128 matched most closely to the loci in Clades 1 and 2 while 1/2a gene 127 matched most strongly with the Clade 5 strain (Table S4 in Supplementary Material). This pattern of genetic loci with differing levels of homology within the group was consistent when 4bV strains were used as the reference for the genomic comparison, with some loci having higher identity to the 4b isolate and others the 1/2a.

Examination of the functional role of these proteins (Table S4 in Supplementary Material), excluding those linked to the 4bV acquired region, reveals that the vast majority (61 of 99) are either hypothetical or phage related. An additional six are putative proteins with no clear function, e.g., lipoprotein, secreted protein, and peptidoglycan bound protein. Seven are internalin or internalin-like proteins. In addition, other genes that may have roles in survival, e.g., ferrous iron transport, were also observed.

## Discussion

Recent listeriosis outbreaks associated with closely related strains of serotype 4bV of Lm may indicate emergence of a new clade or EC (7, 8). Although several 4bV strains have been linked to cases of human listeriosis, the relatedness among these 4bV strains has never been systematically analyzed. This study is the largest to date evaluating the genomic relationships between serotype 4bV strains. While several clades are identified in this study, two in particular, Clades 1 and 2, are notable due to their observed frequency, diversity and geographic dispersal. Examination of food and environmental isolates of Clades 1 and 2 shows that in the USA, Clade 1 strains are predominantly associated with California, while Clade 2 is found generally east of the Mississippi River, suggesting the possibility of geographic bias of these strains.

Studies of Lm have shown that clonal groups are generally globally distributed (30). However, some evidence for geographic distribution has been observed. For example, Zhang et al. observed a divergence in strains of a subgroup based on Chinese and Canadian origination (31). *Clostridium botulinum* has a similar profile with several analyses showing no evidence of geographic bias while others suggest the possibility that certain groups do (32). Other foodborne pathogens not linked to soil, but rather animal hosts, show more clear geographical differentiation as seen in research involving *E. coli* and *Salmonella*, although in the latter case geographic dissemination has been linked to the animal host, e.g., farm to farm spread versus wild birds (33–35).

We employed two methods to evaluate phylogenetic relationships within the 4bV strains studied to rigorously examine phylogeny, WGS SNP analysis and a six gene MVLST. Part of the reasoning for this was an observation in prior work (8) that one strain, LS542 (Ext), exhibited different phylogenies when analyzed by MVLST and WGS SNP analysis. However, given the smaller dataset in that study, we could not determine if this was an outlier or a more significant observation. The six gene MVLST approach used here has been widely shown to agree with MLST typing methods, both of which were used prior to the advent of WGS as a tool for strain discrimination (36). While there was some debate that MVLST would show altered evolution compared to MLST, this was found not to be the case (30, 36).

The results of this study suggest two interesting features related to 4bV strains, relevant to food safety. First, the phylogeny revealed using the six gene MVLST approach suggests the possibility that these loci in 4bV strains undergo different selective pressures, resulting in convergent evolution or reduced divergence of those genes from a theoretical common ancestor. The WGS SNP pipeline analysis supports a different evolutionary history, shown by the seven distinct phylogenetic groups observed *via* the whole-genome analysis, compared to the two MVLST-derived clusters. This observation suggests that virulence genes analyzed in the MVLST scheme in 4bV strains may have been utilized in an alternate fashion when responding to different selection pressure(s) as indicated by their association with atypical foods and a few atypical cases associated with Clade 1 (13–15).

The second feature is that while Clade 2 is a more diverse group, as of yet it does not appear to be responsible for any reported outbreaks on the scale as seen with Clade 1, although a single outbreak occurred in 2014 involving five cases. This is somewhat surprising given that both groups having an equivalent historical presence in the dataset, with a Clade 1 isolate collected as early as 2003 and a Clade 2 isolate as early as 2001. In addition, the presence of smaller 4bV clusters associated with fewer cases suggests the possibility that either Clade 1 strains have higher pathogenic potential or that the members of the other clades have not produced an outbreak due to lower prevalence of the strains resulting in less frequent contamination in sources and only sporadic cases. In the first instance, genetic differences in Clade 1, most likely outside the six virulence loci given the conservation observed in this region between Clade 1 and most of Clade 2, may enhance survival in foods and/or the processing environment or may enhance virulence, leading to increased risk. In fact, differences can be found in the genomic comparisons conducted in this work with several loci uniquely present or absent in the various representative 4bV strains.

Conversely, although strains belonging to Clade 1 and Clade2 are both 4bV Lm and thus should have similar pathogenic potential, differences in agricultural practices, differences among food commodities, and ecological factors may also help explain why only Clade1 strains have been involved in foodborne outbreaks. Identification of these factors and their impact, therefore, becomes critical as they could be used to reduce the risk of future outbreaks for both clades. In this case, the isolates observed in Clade 2 would have the same inherent pathogenic potential and pose a future risk if variables linked to these outbreaks are not identified and controlled. The identification of atypical food vehicles being associated with both of the larger clusters indicates the possibility that 4bV strains have an expanded niche. Overall, the data suggest that 4bV strains of Clade 1 may represent an emerging threat to public health and that this risk may also be applicable to other 4bV strains.

Comparisons between the genomic content of representative 4bV strains to that of a 4b and 1/2a strain, and detailed comparison of the 6.3kb region identified several changes unique to the 4bV isolates further highlighting the possibility of alternate selective pressures leading to an organism that may be better adapted to surviving environmental pressures, compared to typical 4b strains. In addition, the observation of a subset of 4bV strains possessing Lmo0740 proteins that more closely match with strains from environmental lineages is particularly intriguing and suggests the possibility that 4bV strains may represent a hybrid phenotype with clinical and environmental adaptations. Given the conservation of these mutations between this 4bV subset and the lineage III/IV stains, we cannot rule out the possibility of a second transfer event within these strains.

These data suggest that 4bV strains may represent an emerging public health threat, having acquired traits that may enhance survival in environments relevant to food production, transport, and storage and potentially enhance their pathogenic potential. Careful phenotypic and virulence assessments of strains from the various 4bV groups are needed to evaluate whether there are differences in adaptation unique to specific 4bV clusters or if 4bV strains as a whole represent an emerging public health risk.

## Author Contributions

LB, CG, and AD were responsible for the concept of this study and critical revisions of the manuscript. LB and CG were responsible for data collection. LB, CG, and MM contributed to data analysis and interpretation. LB drafted the manuscript. All the authors approved the published version of the manuscript.

## Conflict of Interest Statement

The authors declare that the research was conducted in the absence of any commercial or financial relationships that could be construed as a potential conflict of interest.

## Supplementary Material

Figure S1 A maximum likelihood tree of all strains evaluated in this study derived from SNP alignment file generated using the CFSAN SNP Pipeline with bootstrapping (*n* = 1,000). Strains linked to specific outbreaks are renamed as noted in Table S1 in Supplementary Material for ease of reference. Pipeline clades are highlighted. The scale bar indicates distance as assessed by the Tamura–Nei method.

Figure S2 Maximum likelihood trees of all strains **(A)** and only the serotype 4 (4b, 4d, and 4bV) strains **(B)** evaluated in this study derived from SNP alignment file generated for multi-virulence-locus sequence typing analysis with bootstrapping (*n* = 1,000). Strains linked to specific outbreaks are renamed as noted in Table S1 in Supplementary Material for ease of reference. Previously identified clades and pipeline clades are highlighted where distinct clustering is observed. The scale bar indicates distance as assessed by the Tamura–Nei method.

Table S1 Strains used in this phylogenetic comparison. The file contains accession numbers, strain identifiers, geographic source, and isolation date, where available, serotype and SNP pipeline clade association.

Table S2 The SNP differences matrix generated in the SNP pipeline analysis for all strains evaluated in this study. The table is color coded so that differences less than 10 SNPs are highlighted green, differences of 10–500 are highlighted yellow, differences of 500–4,500 and differences greater than 8,000 are highlighted in blue. Clusters associated with ECI and the 4bV clades are highlighted *via* borders within the table.

Table S3 The SNPs noted in the lmo0733–lmo0739 region when aligning the region from lineage I, II, and III/IV strains, including reference strains (Table 1) and other strains as noted in Table S1 in Supplementary Material. Strains were compared and grouped based on their alignments (data not shown) to select a representative. Each new group represents a single base change from at least one other strain. Row 1 indicates the number of strains represented by the respective column. Row 2 indicates the sequence used as the representative in the alignment. ^a^Indicates the percent of the strains containing the predominant (>50%) lineage I nucleotide at the evaluated location; ^b^Indicates the percent of 4bV strains matching the lineage I predominant nucleotide or, if there is no lineage I nucleotide, the percent of the 4bV strains containing the predominant nucleotide observed in 4bV strains; ^c^Indicates the percent of lineage II strains matching the lineage I strains or 4bV strains if there is no lineage I base. *Indicates predominant nucleotide when <90%.

Table S4 The genomic comparisons *via* RAST of five reference strains evaluated to identify other regions suggesting divergence from the 4b strain. Contig refers to the contig number in the assembly that contains the gene while gene refers to the overall locus number assigned to the ORF. % Identities between the protein sequences with either the 1/2a or the 4b strain as the reference, as noted in the column header. ^a^Three 1/2a loci present in all three 4bV strains but not in the 4b strain; ^b^Two 1/2a loci matching to the same 4bV loci with no orthologs in 4b; ^c^1/2a loci associated with the 6.3 kb island transferred to 4bV strains; alternate, indicates that the loci match differed when using the 4b strain as the reference in the RAST comparison instead of the 1/2a strain.

Data Sheet S1 The SNP alignment FASTA file generated using the CFSAN SNP Pipeline tool. *Indicates files previously available but not currently available.

Data Sheet S2 The SNP alignment FASTA file generated from the six gene multi-virulence-locus sequence typing analysis.
