# Supplementary figures and images for: A Comprehensive Evaluation of the Genetic Relatedness of Listeria monocytogenes Serotype 4b Variant Strains
Source: Front Public Health. 2017 Sep 13;5:241. doi: 10.3389/fpubh.2017.00241 (PMC5601410; doi:10.3389/fpubh.2017.00241)

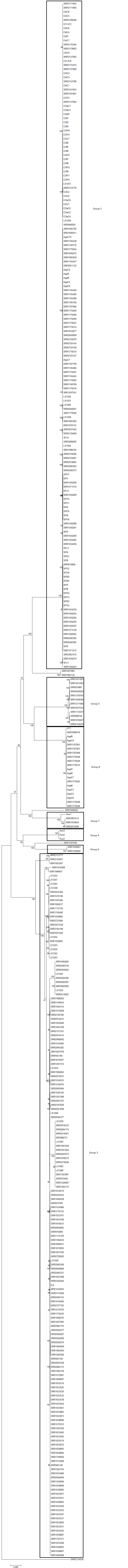

Supplement: Figure S1 — A maximum likelihood tree of all strains evaluated in this study derived from SNP alignment file generated using the CFSAN SNP Pipeline with bootstrapping (n = 1,000). Strains linked to specific outbreaks are renamed as noted in Table S1 in Supplementary Material for ease of reference. Pipeline clades are highlighted. The scale bar indicates distance as assessed by the Tamura–Nei method. [file Image_1.TIF]
